# Gastrointestinal manifestations during oral immunotherapy: A guide for pediatric allergists

**DOI:** 10.1111/pai.70417

**Published:** 2026-07-10

**Authors:** Martina Votto, Roberta Olcese, Maria De Filippo, Lucia Caminiti, Francesco Catamerò, Filippo Favuzza, Simone Foti Randazzese, Massimo Landi, Gian Luigi Marseglia, Michele Miraglia Del Giudice, Mattia Giovannini, Salvatore Barberi

**Affiliations:** ^1^ Maternity and Pediatric Services – Local Health Units Benevento Italy; ^2^ Department of Clinical, Surgical, Diagnostic, and Pediatric Sciences University of Pavia Pavia Italy; ^3^ Allergy Center IRCCS Giannina Gaslini Genoa Italy; ^4^ Department of Maternal, Infantile and Urological Sciences AOU Policlinico Umberto I Rome Italy; ^5^ Pediatric Unit, Department of Human Pathology in Adult and Developmental Age “Gaetano Barresi” University of Messina Messina Italy; ^6^ Allergy Unit Meyer Children's Hospital IRCCS Florence Italy; ^7^ Department of Health Sciences University of Florence Florence Italy; ^8^ Pediatric Unit Hospital Holy Family Fatebenefratelli Company Erba Italy; ^9^ ENT and Allergy Unit Humanitas Cellini Clinical Institute Turin Italy; ^10^ Pediatric Clinic Fondazione IRCCS Policlinico San Matteo Pavia Italy; ^11^ Department of Woman, Child and General and Specialized Surgery University of Campania ‘Luigi Vanvitelli’ Naples Italy; ^12^ Pediatric Unit ASST‐Rhodense, RHO Milan Italy

**Keywords:** anaphylaxis, eosinophilic esophagitis, food allergy, gastrointestinal manifestations, oral immunotherapy, oral immunotherapy‐related gastrointestinal and eosinophilic responses

## Abstract

Oral immunotherapy (OIT) is a promising therapeutic strategy for desensitizing patients with immunoglobulin E (IgE)‐mediated food allergies. Although effective, OIT is frequently associated with adverse events (AEs), and gastrointestinal (GI) manifestations are among the most common and challenging AEs, which are the leading cause of OIT discontinuation. These reactions range from immediate IgE‐mediated signs and symptoms to delayed, non‐IgE‐mediated, eosinophil‐associated disorders, including eosinophilic esophagitis (EoE). While systemic reactions, such as anaphylaxis, are well‐known concerns, GI manifestations are frequently encountered, although they are often less severe than systemic reactions. A thorough understanding of GI signs and symptoms is therefore essential for patients and clinicians to navigate OIT effectively. This narrative review provides a comprehensive overview of GI AEs observed during OIT, detailing their clinical presentation, underlying pathophysiology, and evidence‐based management approaches. A clear understanding of these manifestations and their management is essential for optimizing patient safety and treatment adherence in the era of OIT.

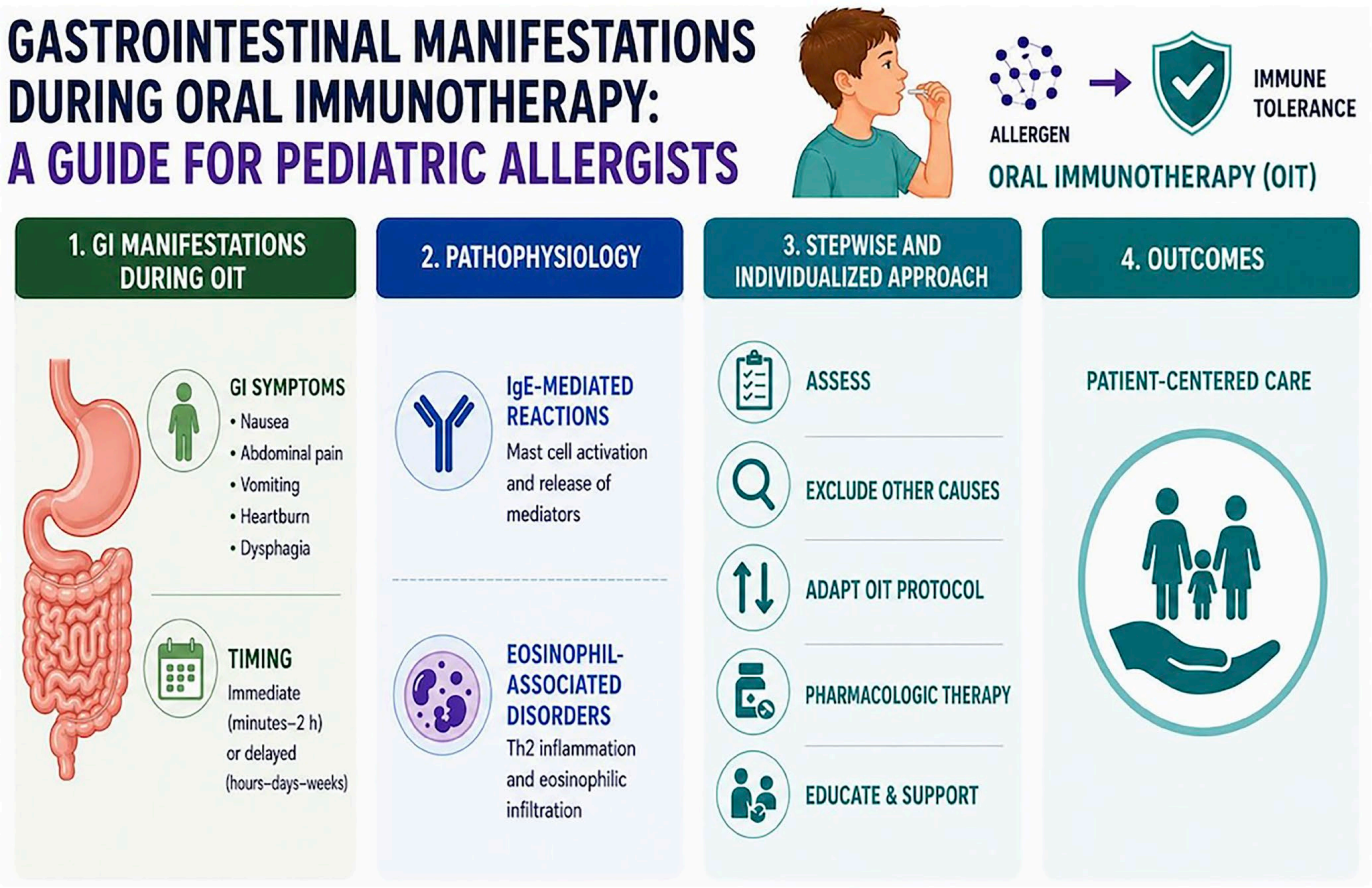

## Graphical abstract

Gastrointestinal manifestations occurring during oral immunotherapy (OIT) encompass a spectrum of conditions ranging from immediate IgE‐mediated reactions to delayed eosinophil‐associated disorders, including eosinophilic esophagitis. Because symptoms may overlap with other gastrointestinal diseases, a structured assessment based on symptom timing, clinical features, and differential diagnosis is essential. Individualized management strategies, including dose adjustment, pharmacological treatment, endoscopic evaluation when indicated, and shared decision‐making with patients and families, may help optimize treatment adherence while maintaining safety during OIT.

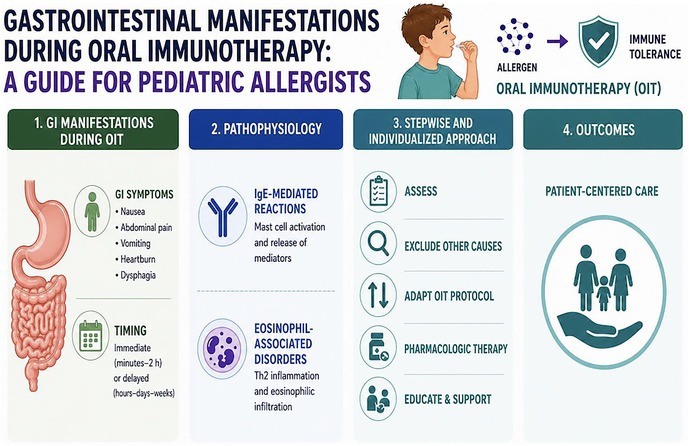

**Key message**

OIT is a promising therapeutic strategy for treating food allergy. However, OIT is often associated with GI manifestations, which are the most common and challenging AEs and the leading cause of OIT discontinuation. These reactions range from immediate IgE‐mediated symptoms to delayed, non‐IgE‐mediated, eosinophil‐associated disorders, including EoE. A shared decision‐making approach, guided by symptom severity and patient priorities, is essential for managing GI AEs. This approach combines dose adjustments with appropriate diagnostic workups, including endoscopy, and targeted pharmacological treatment.

## 1 INTRODUCTION

Immunoglobulin E (IgE)‐mediated food allergy (FA) is a growing global issue affecting people of all ages, especially children.^1^ FA and food‐induced anaphylaxis significantly impact patients' quality of life (QoL) and healthcare systems.^2^, ^3^ Approximately 7 million Europeans live with food allergies, with 8% experiencing a life‐threatening reaction. In the US, FA affects approximately 33 million people and 8% of the pediatric population.^3^, ^4^, ^5^ FA signs and symptoms can involve multiple systems and organs, ranging from mild clinical manifestations (e.g., localized urticaria) to severe, life‐threatening conditions, including anaphylaxis and anaphylactic shock.^6^ The cornerstone of FA treatment is avoiding the culprit food(s), which exposes the patients to the potential risk for nutritional deficiencies, growth issues, and reduced QoL.^7^, ^8^ Additionally, food avoidance does not alter the natural course of the disease and the underlying cause.^9^ As a result, patients may experience allergic reactions when unintentionally exposed to the culprit food(s).

Allergen immunotherapy (AIT) is the only approved treatment that induces immunotolerance in allergic patients.^9^ AIT aims to retrain the immune system to tolerate allergenic foods rather than overreact to them, marking a significant shift from traditional management, which focuses solely on strict avoidance and emergency medication. The core principle involves controlled, gradual exposure to increasing amounts of the allergen, allowing the body to build a protective immune response.^10^ Different AIT administration routes are actively being researched and implemented for the treatment of FA.^9^, ^10^ Sublingual immunotherapy (SLIT) involves placing small amounts of the allergen extract under the tongue. Although it is generally considered safe due to lower systemic exposure, the efficacy of SLIT for FA is often less robust than that of the oral route.^11^ Epicutaneous immunotherapy (EPIT) is an innovative approach that delivers the allergen through a patch applied to the skin. The allergen is absorbed through the skin and presented to the immune system of the host. EPIT has gained attention, particularly for peanut allergy, with the commercial availability of a patch that offers a new treatment option.^11^, ^12^

**FIGURE 1:**
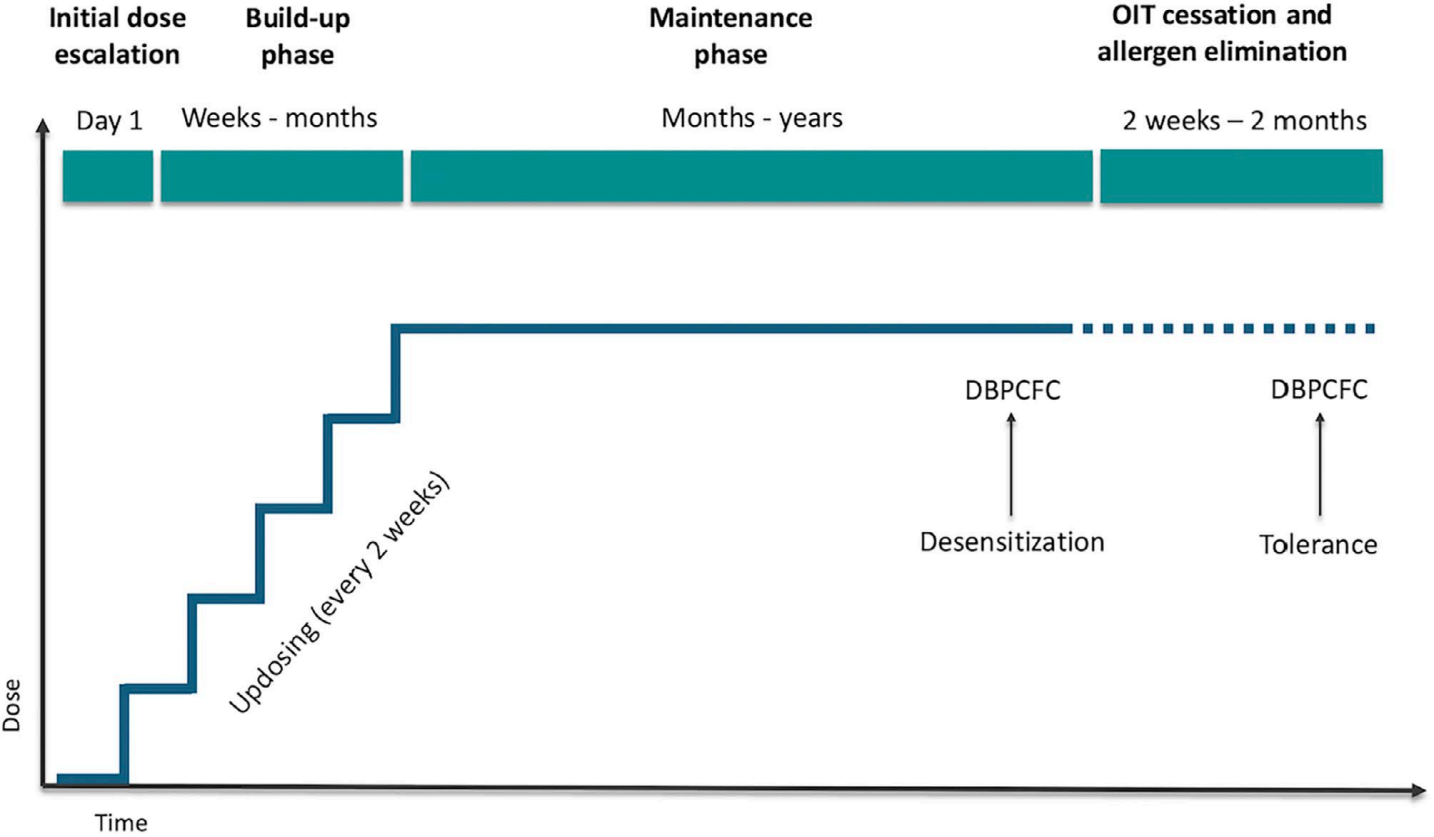
A typical OIT protocol (adapted from Gernez and Nowak‐Węgrzyn).^17^ The initial phase of treatment, typically lasting 1–2 days, involves rapid up‐dosing. This process begins with a very small dose, which is unlikely to cause an adverse reaction, and is gradually increased to a dose that is considered safe for home administration. The doses start at microgram quantities of the allergenic protein and increase to several milligrams by the end of this phase. If a patient tolerates the initial dose, the dose is increased incrementally, usually on a biweekly or weekly basis. This continues until a target maintenance dose is achieved or the patient experiences dose‐limiting symptoms. The target maintenance dose varies significantly across studies, ranging from 300 mg to 4000 mg. Maintenance therapy involves daily home administration and can last for months to several years, as consistent exposure is crucial to sustaining immune system changes. After a prolonged maintenance period, allergists may consider assessing sustained unresponsiveness. This involves temporarily stopping daily allergen intake for a defined period (e.g., 2 weeks to 1 year), followed by an oral food challenge. Successfully passing this challenge indicates that the individual can tolerate the allergen even after treatment cessation, signifying a more durable form of tolerance. DBPCFC, Double blind placebo‐controlled food challenge.

Oral immunotherapy (OIT) is the most widely studied and implemented form of AIT and has demonstrated significant success, particularly for peanut, milk, and egg allergies.^13^, ^14^ An approved oral peanut product is now available for clinical use.^15^, ^16^ The benefits of OIT are substantial. Patients can experience a dramatic decrease in anxiety about accidental exposures, improved QoL, and, in most cases, the ability to reintroduce foods they previously had to avoid. Patient selection for OIT is critical, and factors such as allergy severity, potential comorbidities, and the commitment of the patient and family to the demanding protocol should be considered. The OIT protocol is highly structured and typically involves distinct phases, all conducted under close medical supervision to ensure patient safety (Figure 1).^17^, ^18^ Despite the high success rate in developing tolerance, OIT compliance is limited by frequent side effects that often occur during the initial or up‐dosing phases of treatment.^19^ Allergic reactions are a typical and expected part of the OIT process. They can range from mild local symptoms, such as oral itching, abdominal discomfort, and hives, to more moderate or severe systemic reactions, including anaphylaxis. The most common and relevant side effects involve the gastrointestinal (GI) system and can occur at different stages of the OIT protocol.^20^ The underlying pathogenic mechanisms vary and are not always related to allergen dose. GI manifestations can range from IgE‐mediated reactions to non‐IgE‐mediated conditions, including eosinophilic esophagitis (EoE).^19^, ^20^, ^21^, ^22^ Recognizing these potential side effects is essential for the proper management and continuation of the desensitization protocol.

This narrative review aims to provide an accessible overview of OIT and its associated GI manifestations, with a particular focus on delayed, non‐IgE‐mediated GI symptoms, including their prevalence and management strategies.

## 2 METHODS

A literature search was conducted in July 2025 using the PubMed database. The search terms combined the Medical Subject Headings (MeSH) terms “oral immunotherapy” and “eosinophilic esophagitis” or “OITIGER” (OIT‐related gastrointestinal and eosinophilic responses) or “ELORS” (eosinophilic esophagitis‐like oral immunotherapy‐related syndrome). Articles were selected for relevance to delayed GI outcomes (OITIGER, ELORS, and EoE) during OIT. Eligible studies included systematic reviews, meta‐analyses, trials, and observational studies reporting the prevalence of delayed GI symptoms following OIT, published in English in peer‐reviewed journals. No strict restrictions were applied to study design, in line with the narrative review approach. The reference lists of the included articles were screened to identify additional relevant studies. The selected literature was qualitatively synthesized to provide an informative and educational overview of GI manifestations, their prevalence, clinical presentation, and management during OIT.

## 3 GASTROINTESTINAL MANIFESTATIONS DURING ORAL IMMUNOTHERAPY

GI manifestations are common during OIT, affecting a large proportion of patients undergoing OIT.^23^, ^24^ The overall rate of GI manifestations leading to OIT discontinuation has been reported to range from 10% to 20%.^23^ GI manifestations are difficult to define because they can occur in two distinct clinical situations during OIT. In the first case, GI symptoms appear rapidly, within minutes after dose administration, as a result of an immediate IgE‐mediated reaction. In the second scenario, GI symptoms may be delayed and associated with non‐anaphylactic eosinophil‐related GI adverse events (AEs). Therefore, GI manifestations can be broadly categorized by their underlying immunological mechanisms (immediate or IgE‐mediated; delayed or non‐IgE‐mediated/eosinophil‐driven), each requiring distinct diagnostic and therapeutic approaches.

IgE‐mediated reactions are typically acute, often occurring within 2 h of exposure to the causative allergen. In type I immune reactions, the allergen binds to IgE antibodies on the surface of mast cells and basophils in the GI mucosa, triggering degranulation and the release of inflammatory mediators, such as histamine. This results in signs and symptoms such as oral pruritus, nausea, vomiting, and abdominal pain, as well as systemic reactions, including anaphylaxis. IgE‐mediated GI manifestations are common and generally expected during OIT.^22^

In 2017, Goldberg et al., analyzing data from 794 patients undergoing OIT for different food allergens (milk, peanut, sesame, or egg), reported that approximately 8% developed recurrent episodes (≥3/month) of GI manifestations with blood eosinophilia, independent of the timing of dose administration (Table 1).^25^ The authors named this constellation of symptoms “OIT‐related gastrointestinal and eosinophilic responses” (OITIGER). This GI condition generally occurred during the build‐up phase, within weeks to several months after exposure to the culprit antigen, and was characterized primarily by recurrent abdominal pain, nausea, and vomiting. Only three patients underwent esophagogastroduodenoscopy (EGD), which revealed a pathologic increase in tissue eosinophils, suggesting EoE. For the other patients, signs and symptoms resolved with a reduction in OIT dose. The authors also found that patients with these signs and symptoms exhibited peripheral eosinophilia, which significantly decreased after dose reduction, suggesting that blood eosinophil count may be a potential marker of GI involvement during OIT. After dose reduction, more than 90% of patients who resumed treatment did not experience a recurrence of GI manifestations.^25^, ^29^

**TABLE 1:** Summary of relevant studies reporting the prevalence of non‐IgE‐mediated GI manifestations during OIT.

References	Delayed GI manifestation	Allergen(s)	Prevalence (%)	Study
Lucendo et al.^24^	Biopsy‐confirmed EoE	Milk, peanut, and egg	2.7%	Systematic review with meta‐analysis
Goldberg et al.^25^	OITIGER	Milk, peanut, and egg	8.2%	Observational study
Petroni and Spergel^26^	EoE	Milk, peanut, and egg	5.3%	Systematic review
Wasserman et al.^27^	ELORS	Peanut	13.7%	Observational study
Rossi et al.^28^	EoE	Milk, peanut, and egg	2.3%	Systematic review with meta‐analysis

Abbreviations: ELORS, eosinophilic esophagitis‐like oral immunotherapy‐related syndrome; EoE, eosinophilic esophagitis; OIT, oral immunotherapy; OITIGER, OIT‐related gastrointestinal and eosinophilic responses.

In a study of peanut OIT, Wasserman et al. reported similar findings. They retrospectively reviewed the medical records of 270 consecutive patients who received peanut OIT between 2009 and 2017. During the OIT protocol, mainly during the escalation phase, 37 patients (14%) experienced episodic GI manifestations (especially vomiting) occurring more than 2 h after dosing. The authors termed these manifestations “eosinophilic esophagitis‐like oral immunotherapy‐related syndrome” (ELORS). Thirteen patients (35%) also had an increase in peripheral blood eosinophil counts. OIT was continued with dose reduction in 21 patients; only five subjects discontinued the peanut desensitization protocol due to GI manifestations. Eighteen patients with ELORS were treated with short courses (1–4 weeks) of proton pump inhibitors (PPIs), and one received topical corticosteroids for less than 14 days. None had persistent signs and symptoms or required prolonged therapy.^27^

The first case of EoE occurring during OIT for peanut allergy was documented in 2009.^20^ Since then, newly confirmed EoE diagnoses have been reported as an adverse event of OIT in approximately 2.7%–5.3% of patients, with a 5.6% OIT discontinuation rate due to EoE or symptoms possibly related to it.^19^, ^20^ More recently, Rossi et al., in a systematic review with meta‐analysis, reported that the EoE incidence during OIT is 2.31% (95% CI 1.45, 3.36).^28^ EoE is a chronic, immune‐mediated inflammatory disease of the esophagus, characterized by eosinophil‐predominant infiltration of the esophageal mucosa.^30^, ^31^ First described as a distinct clinicopathological entity in the early 1990s, EoE is now recognized as a significant cause of esophageal morbidity and a growing health concern. Its pathogenesis involves a complex interplay among genetic predisposition, epithelial barrier damage, environmental and food allergen exposure, esophageal dysbiosis, and immunological pathways, primarily mediated by a T helper type 2 (Th2) inflammatory response.^32^ The disease is strongly linked to atopy and has a high comorbidity rate with allergic rhinitis, asthma, and atopic dermatitis.^33^ The clinical presentation of EoE varies by age and is often nonspecific. In adults, common signs and symptoms include dysphagia, especially for solid foods, and food impaction, which may require emergency endoscopic removal. Pediatric patients often present with subtler symptoms such as feeding difficulties, vomiting, abdominal pain, and failure to thrive. EoE can also present with less specific symptoms that affect eating habits or with eating disorders in adolescents and young adults.^31^, ^34^ The current diagnostic criteria for EoE include signs and symptoms suggestive of esophageal dysfunction and an esophageal biopsy showing a peak eosinophil count of ≥15 eosinophils per high‐power field (eos/HPF). Effective management of EoE is vital to prevent long‐term complications such as esophageal strictures and fibrosis. Treatment strategies are multimodal, combining food elimination diets, pharmacologic therapies (such as PPIs, topical corticosteroids, and anti‐IL‐4/−13 biological agents), and endoscopic interventions.^35^

Although similar, OITIGER and ELORS differ in several clinical features. Both conditions occur during the early stages of the OIT protocol, are dose‐dependent, and resolve with a reduction in OIT dose. While OITIGER is characterized by multiple episodes of nonspecific, delayed abdominal pain, ELORS is primarily marked by vomiting that can occur 2–6 h after the OIT dose and is not necessarily associated with epigastric pain or nausea. Peripheral blood eosinophilia is observed in most cases of both conditions, but it is more pronounced in OITIGER, in whom the eosinophil count can exceed 0.9 × 10^9^ cells/L (Table 2).^25^, ^27^ Whether ELORS and OITIGER represent the same disease remains uncertain, but they should currently be considered distinct diseases on the same spectrum.^21^

**TABLE 2:** Clinical features, differential diagnosis, and management of delayed GI manifestations during OIT.

	Immediate GI manifestations	Delayed GI manifestations		
	IgE‐mediated reactions	OITIGER	ELORS	EoE
Time of onset	Minutes (within 2 h) after taking the dose	Not temporally related to dose administration. >2 h after taking the dose	Not temporally related to dose administration. 2–6 h after taking the dose	Not temporally related to dose administration
Phase of OIT protocol	Initial dose escalation and build‐up phases	Early phases (weeks after starting the protocol)	Early phases (weeks after starting the protocol)	Maintenance phase
Sign and symptoms	Mild–moderate (hives, itch, abdominal pain, vomiting) to severe and systemic (anaphylaxis, airway angioedema, acute asthma attack, anaphylactic shock)	At least 3 episodes of abdominal pain (main symptom) and vomiting in a month + increase in blood eosinophilia (>900 cells/mm^3^) and sometimes non EoE esophageal eosinophilia.	Recurrent vomiting (main symptom), epigastric abdominal pain or nausea + increase in blood eosinophilia (less common than OITIGER)	Persistent GI symptoms >4 weeks and depending by the age. The most common symptoms are GERD‐like symptoms, vomiting, food refusal and changes in eating behaviors, dysphagia, food impaction, epigastric pain
Differential diagnosis	Anxiety‐related symptoms, concomitant infections	Acute gastroenteritis, GERD, functional GI disorders, intestinal parasitosis, EGIDs	Acute gastroenteritis, GERD, functional GI disorders, intestinal parasitosis, EGIDs	GERD, infectious esophagitis, IBD, celiac disease, achalasia, HES, vasculitis
Natural course	Worsening with dose increases and gradual improvement over time on dose	Transient	Transient	Often reversible after OIT discontinuation. However, there are cases of EoE that not improved with OIT discontinuation
Management	Antihistamines, systemic steroids; IM epinephrine in case of anaphylaxis	EGD generally not necessary. OIT dose reduction, Slower dose escalation, Short courses of PPIs (8–12 weeks).	EGD generally not necessary. OIT dose reduction, Slower dose escalation, Short courses of PPIs (8–12 weeks).	EGD for confirmation. Shared decision‐making approach. PPIs or topical corticosteroids, OIT discontinuation, Consider switching to GIDOIT.

Abbreviations: AEs, adverse events; EGIDs, eosinophilic gastrointestinal disorders; ELORS, eosinophilic esophagitis‐like oral immunotherapy‐related syndrome; EoE, eosinophilic esophagitis; GERD, gastroesophageal reflux disease; GI, gastrointestinal; GIDOIT, gastrointestinal delivery oral immunotherapy; HES, hypereosinophilic syndrome; IBD, inflammatory bowel disease; OIT, oral immunotherapy; OITIGER, OIT‐related gastrointestinal and eosinophilic responses; PPIs, proton pump inhibitors.

OITIGER, ELORS, and EoE share similar histological findings and likely, Th2 and eosinophil‐driven pathogenic mechanisms. These eosinophil‐associated diseases are a more complex and persistent group of OIT‐related manifestations that often occur weeks or months after treatment initiation.^23^ They are characterized by significant eosinophil infiltration into the GI tract, particularly the esophagus. The exact pathogenetic mechanisms are still under investigation, but they are thought to involve a persistent Th2 inflammatory response that fails to resolve and may be influenced by a preexisting predisposition to atopic diseases.^23^ During OIT, repeated exposure to allergens in the esophageal lumen triggers the release of alarmins from the epithelium, such as thymic stromal lymphopoietin (TSLP), interleukin (IL)‐33, and IL‐25. These signals activate Th2 cells, which release IL‐4, IL‐5, and IL‐13, and innate lymphoid cells (ILCs), which contribute to eosinophilic influx independently of traditional T‐cell pathways. Notably, IL‐13 plays a central role in upregulating eotaxin‐3 transcription in esophageal epithelial cells, which acts as a potent chemoattractant for eosinophils via the CCR3 receptor. Moreover, IL‐13 promotes barrier disruption by reducing desmoglein 1 (DSG1) expression and weakening the desmosomes that hold epithelial cells together. In a vicious cycle, continuous allergen contact during OIT may exacerbate the underlying dysfunction of the esophageal barrier.^36^ Interestingly, OIT significantly increases allergen‐specific IgG4 production, which reflects immunotolerance mechanisms and is protective against specific IgE. Some studies have suggested that IgG4 deposits are present in the esophageal tissue of EoE patients, although their role as drivers of inflammation versus markers of exposure remains debated.^36^

However, a pathogenic link between OITIGER or ELORS and EoE has not yet been demonstrated. In reported studies, EGD was generally not performed because signs and symptoms were treated and resolved clinically, and diagnostic biopsies were not always obtained. EoE is rarely observed during the early phases of the OIT protocol. Wright et al. evaluated preexisting esophageal eosinophilia in adults with IgE‐mediated peanut allergy before desensitization. Preexisting esophageal eosinophilia with clinical manifestations (≥5 eos/HPF) was present in five participants (24%), three (14%) of whom had ≥15 eos/HPF, consistent with a diagnosis of EoE.^37^ Subsequently, in a randomized, placebo‐controlled trial, the same research group found that OIT induced or exacerbated esophageal eosinophilia in most peanut OIT patients at week 52, which resolved by the end of the maintenance phase. Only one patient developed EoE and withdrew from the study. Interestingly, EoE did not occur in subjects receiving a placebo. Active treatment with OIT usually induced transient esophageal eosinophilia, which may meet EoE diagnostic criteria but typically resolved over time.^38^ Subsequently, Avinashi et al. reported the case of a 12‐year‐old boy who developed esophageal strictures after only 3 weeks of peanut OIT, which are typical endoscopic lesions related to persistent or untreated eosinophilic inflammation.^39^ In this context, Duman Senol et al. recently demonstrated that asymptomatic esophageal eosinophilia is more common than previously thought in food‐allergic children. They enrolled 48 asymptomatic children (3–15 years) who underwent EGD before OIT. Pathologic endoscopic findings were present in 75% of patients, including sixteen (33%) with esophageal eosinophilia and five (10%) with low‐grade eosinophilia (5–15 eos/HPF).^40^ These findings provide pediatric data on preexisting esophageal eosinophilia before OIT, complementing the adult data reported by Wright et al., who demonstrated esophageal eosinophilia in adults with IgE‐mediated food allergy before desensitization.^37^, ^40^ The relationship between OIT‐induced GI manifestations and preexisting, potentially subclinical, EoE is a significant area for further investigation. It remains unclear whether esophageal eosinophilia can progress to EoE without OIT or whether chronic exposure to the food allergen may exacerbate preexisting eosinophilic inflammation.^19^ Therefore, to clarify whether patients undergoing OIT have an inherent susceptibility to EoE, extensive prospective studies, including EGD with biopsies (or less invasive tools such as the string test) before OIT and during the phase characterized by clinical manifestations, are necessary.

Rarely, patients undergoing OIT can also develop non‐EoE eosinophilic gastrointestinal disorders (EGIDs), including eosinophilic gastritis (EoG), enteritis (EoN), and colitis (EoC). Although no studies have assessed the rate of non‐esophageal EGIDs in patients undergoing OIT, anecdotal case reports have been described.^20^ In a study of 128 allergic patients undergoing OIT, eight (6.25%) developed EGID, including two diagnosed with eosinophilic gastroenteritis after initiation of OIT. In these cases, OIT was discontinued because of extensive GI involvement, with clinical improvement.^41^

## 4 MANAGEMENT OF GI MANIFESTATIONS DURING OIT

Diagnosis and treatment of immediate GI manifestations are straightforward and follow the same principles as for IgE‐mediated hypersensitivity reactions.^42^ Oral pruritus and tingling are the most common initial clinical manifestations and are often self‐limiting or easily managed with oral antihistamines. Nausea, vomiting, and abdominal pain can also occur shortly after dosing and are treated with antihistamines. Mild clinical manifestations, such as oral pruritus, can often be managed by reducing the OIT dose or taking an oral antihistamine before the OIT dose. Although IgE‐mediated signs and symptoms are generally mild and tend to decrease with successful desensitization, they can also be associated with systemic anaphylactic reactions. For severe or systemic clinical manifestations (diffuse mucocutaneous + GI symptoms, airway angioedema, or collapse), epinephrine should be administered intramuscularly, and the patient should be evaluated in an emergency setting.^41^, ^42^

Several clinical factors make the diagnosis of OIT‐related GI AEs critical, as symptoms are nonspecific or elusive and no validated biomarkers are sufficiently sensitive to replace upper GI endoscopy (Table 2).^42^, ^43^ GI symptoms are common in children and often nonspecific, particularly abdominal pain and vomiting.^31^ Changes in eating habits and feeding issues develop gradually in the early stages of EoE and are frequently not adequately recognized by caregivers, delaying diagnosis.^31^, ^34^ Young children cannot clearly report their symptoms and may manifest discomfort through irritability or loss of appetite.^30^, ^31^ Subacute or chronic signs and symptoms, whether related or unrelated to OIT, may reflect a broad range of GI disorders, including EGIDs, OITIGER, ELORS, gastroesophageal reflux disease (GERD), celiac disease (CD), functional GI disorders (FGID), or GI infections. Children undergoing OIT can develop other non‐allergic GI diseases, including FGID, GERD, and CD. These manifestations are unrelated to OIT and should be distinguished from those potentially associated with OIT, including OITIGER, ELORS, and EoE.

OITIGER and ELORS typically present early during OIT and can persist for more than 7 days. ELORS generally presents with vomiting 2–6 h after OIT and is not necessarily associated with epigastric pain or nausea. OITIGER is characterized by multiple episodes of abdominal pain. In both entities, an elevated peripheral blood eosinophil count is often reported.^42^ In this context, blood eosinophilia can also serve as a marker of potential eosinophilic inflammation and increase the suspicion of these two OIT‐related disorders compared with other GI diseases. Peripheral blood eosinophilia greater than 900 cells/mm^3^ may support the diagnosis and help monitor treatment responses.^21^ However, this marker is nonspecific and commonly observed in other pediatric conditions, including intestinal parasitosis or moderate to severe atopic dermatitis. When abdominal pain and vomiting occur with other acute signs and symptoms, such as fever and diarrhea, and last for days, infective gastroenteritis is a likely diagnosis. Contact with other individuals who are sick can also help make a precise diagnosis. A viral or bacterial infection is a known cofactor for anaphylaxis during OIT; in such cases, it is necessary to temporarily halt or reduce OIT doses.^42^

Generally, EoE occurs during the maintenance phase of OIT. However, some cases of EoE have been reported after a few weeks of OIT.^20^, ^28^ Given the ethical limitations of EGD, especially in children, assessing for maladaptive eating behaviors, eating disorders, or more suggestive signs and symptoms of esophageal diseases before and during OIT could help identify patients with a suspicious diagnosis of EoE (Table 3).^44^ Although there are overlapping signs and symptoms of EoE and other GI conditions, EoE also presents with unique signs and symptoms, including difficulty swallowing, slow eating, the need to lubricate food with liquids, avoidance of certain foods and medications, and food impaction.^30^, ^31^

**TABLE 3:** Screening questions to be answered before starting and during OIT (adapted from Muir et al.,).^43^

Screening questions	
Toddlers and young children	Children and adolescents
Does the food get stuck in throat when the child eats? Does the child chew a lot? Does the child refuse solid foods? Does the child prefer creamy, soft or liquid foods? Does the child need to drink during the meal? Does the child choke during meals? Does the child have failure to thrive or weight loss? Does the child have a GER not responsive to conventional treatments?	Does the food get stuck in your throat when you eat? Do you have trouble of swallowing? Does it take longer than others to eat? Do you need to cut food into small pieces? Do you need to drink with meals? Do you eat steak? Do you eat crusty bread? Do you need to make the crusty bread softer? Do you need to cut a steak into small pieces? Do you have to get reminded to chew a lot? Are you able to take pills?^a^ Do you have recurrent epigastric/abdominal pain? Do you have GER symptoms not responsive to treatments? Do you have chest pain after meals? Did you lose weight?

Abbreviation: GER, gastroesophageal reflux.

^a^ Especially in adults.

**FIGURE 2:**
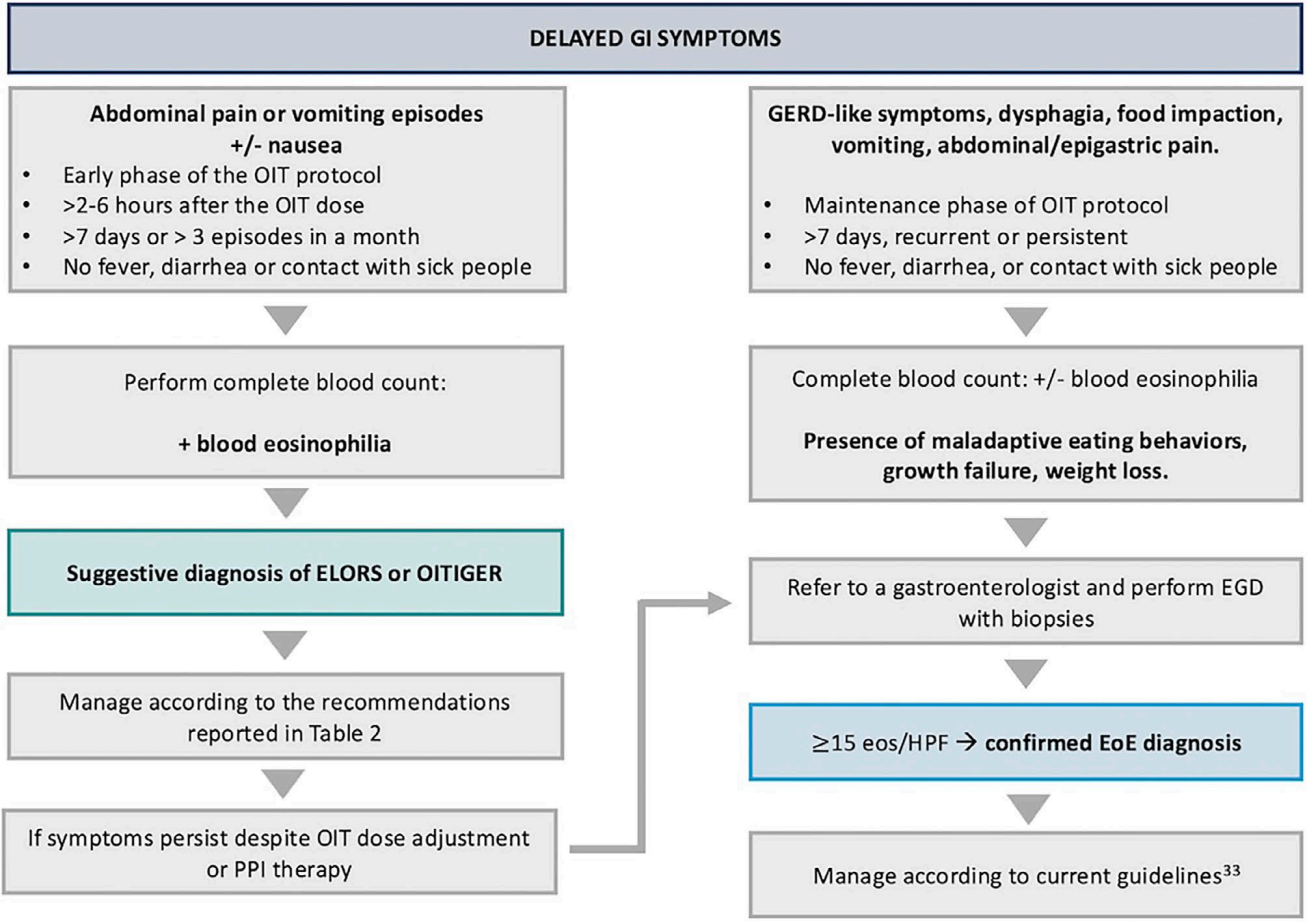
Proposed algorithm for diagnosing delayed GI manifestations during OIT. ELORS, Eosinophilic esophagitis‐like oral immunotherapy‐related syndrome; EoE, Eosinophilic esophagitis; GERD, Gastroesophageal reflux disease; GI, Gastrointestinal; OITIGER, OIT‐related gastrointestinal and eosinophilic responses; OIT, Oral immunotherapy.

EGD with biopsy is the gold standard for diagnosing EoE. It is an invasive procedure that requires general anesthesia in children.^31^, ^34^ The optimal timing of EGD in children with delayed GI symptoms during OIT remains uncertain, constrained by limited evidence and ethical considerations. Moreover, many patients with delayed GI manifestations during OIT are managed clinically, and EGD with biopsies is generally not performed.^42^ The diagnoses of ELORS and OITIGER are generally clinical, based on peripheral blood eosinophilia, early recurrence of GI symptoms, and responsiveness to OIT dose adjustment. However, GI endoscopy with biopsy should also be considered and performed in patients with delayed GI symptoms and blood eosinophilia refractory to dose reduction or PPI therapy for at least 8–12 weeks. In some patients who underwent endoscopic assessment for both early‐ and late‐onset GI manifestations related to OIT, histological findings were consistent with EoE.^28^ In the cohort of 65 patients with OITIGER reported by Goldberg et al., esophageal biopsies were performed in three more severe cases (5%), and each showed more than 15 eosinophils per high‐power field, consistent with EoE.^24^ Persistent or recurrent symptoms suggestive of esophageal dysfunction should raise suspicion for EoE, and patients should be promptly referred to a gastroenterologist for endoscopic evaluation.^42^ Given the broad spectrum of potential, sometimes overlapping diagnoses, a standardized, stepwise approach is crucial for managing GI manifestations during OIT to ensure patient safety and maximize the likelihood of successful desensitization (Figure 2).

Managing delayed GI manifestations is challenging and hampered by limited evidence.^42^ OITIGER and ELORS are transient manifestations related to the initial high OIT dosing.^25^, ^27^ Most cases of OITIGER and ELORS have been managed with OIT dose reduction and slower subsequent dose escalation.^21^ Goldberg et al. reported that about 30% of patients temporarily discontinued OIT until their symptoms resolved. Once clinical recovery was achieved and the absolute peripheral eosinophil count had normalized, OIT was restarted at a lower dose and gradually increased, with good tolerance. OIT was maintained in 60% of patients, although the total daily cumulative dose was reduced. This reduction was accomplished by lowering the dose, slowing the rate of escalation, and/or decreasing the frequency of administration.^25^ In the study by Wasserman et al., therapy was maintained in 21 patients with ELORS, and 13 of them successfully reached their target dose during dose escalation. Of the eight patients who discontinued treatment, five did so due to ELORS, two for unrelated reasons, and one transferred care after successfully resuming escalation.^27^ Considering their transient natural course and mild‐to‐moderate GI symptoms, patients who develop OITIGER or ELORS should continue OIT at adjusted lower doses to guarantee the tolerance achievement and reduce the risk of severe immediate allergic reactions.

In the event of an EoE diagnosis during OIT, most experts recommend discontinuing the desensitization protocol.^20^, ^21^, ^41^ Evidence generally supports discontinuation of OIT upon confirmation of EoE, particularly in the presence of clinically significant or persistent symptoms and rings and strictures at endoscopy.^42^ However, emerging studies suggest that patients who develop EoE during OIT can be managed with medical therapy and adjustment of OIT dosage.^42^ More recently, a case series involving 13 patients with a newly diagnosed EoE showed that pharmacological therapy, mainly high‐dose PPI, can be effective even without discontinuing OIT.^45^ Some patients with endoscopically confirmed EoE chose to continue OIT while treated with PPIs or swallowed steroids to derive a greater benefit from OIT in preventing anaphylaxis from accidental exposure. Conversely, some patients decided to stop OIT permanently due to GI manifestations or concerns about potential long‐term sequelae from EoE.^41^, ^42^, ^46^

Most data supporting management strategies such as dose reduction, slowing up‐dosing schedules, or temporarily interrupting OIT come from observational cohort studies and case series, reflecting the absence of high‐quality comparative trials directly evaluating continuation versus discontinuation of OIT or PPIs versus topical steroids.^25^, ^27^, ^28^, ^45^ The option to stop or continue OIT should be discussed with the patient and their family, and the risks and benefits of each option should be explained through a shared decision‐making approach.^41^ Moreover, continuing OIT in combination with pharmacological therapy should be performed under close clinical and endoscopic monitoring.

Regarding timing, there is insufficient evidence and no universally agreed‐upon timeframe for discontinuing OIT in patients with EoE who choose to continue immunotherapy. In general, pragmatic recommendations suggest clinical and endoscopic reassessment after approximately 8–12 weeks of targeted EoE therapy. For all patients with EGID, allergists should work closely with gastroenterologists to determine the frequency of reassessment endoscopies, which may depend on disease severity and local availability of specialist resources.

The current management of EoE as a side effect of OIT remains highly controversial and requires extensive study before it can be universally and routinely recommended. The management recommendations proposed in this narrative review are largely based on and extrapolated from expert opinion, case reports, and case series, and are also influenced by methodological limitations, including potential selection bias in the literature reviewed. Future recommendations for OIT management in patients who develop EoE should adopt an individualized, shared decision‐making approach that integrates disease severity, therapeutic response, and immunotherapy goals, rather than a rigid, one‐size‐fits‐all algorithm.

## 5 NEW CHALLENGES AND FUTURE DIRECTIONS

In recent years, OIT has been proposed as a treatment for FA in children aged 6 months to 4 years.^22^ While this developmental window offers extraordinary opportunities for desensitization, it also poses unique prognostic challenges that warrant careful consideration. Although the risk of anaphylaxis during dose escalation is well documented, early introduction raises concerns about delayed, more insidious complications, including EoE. In toddlers and preschool‐aged children, diagnosing EoE is challenging because patients often cannot verbalize their symptoms or present with nonspecific GI manifestations.^30^, ^31^ A delayed EoE diagnosis can lead to tissue remodeling and fibrosis, food refusal, avoidant/restrictive food intake disorder (ARFID), failure to thrive, or growth impairment.^34^ To mitigate these risks, recent evidence suggests a precision medicine approach centered on growth monitoring, early screening for maladaptive eating habits, flexible OIT dosing, and multidisciplinary collaboration.^22^, ^47^

Future strategies to manage delayed GI manifestations may include alternative immunotherapy routes, such as EPIT, or delivery methods that bypass the upper GI tract.^28^ GI delivery oral immunotherapy (GIDOIT) is a specific OIT approach for FA, particularly peanut allergy, which involves administering the allergen in sealed capsules to bypass the upper GI tract. Studies have shown that GIDOIT is effective in achieving desensitization to peanuts in patients with FA and in reducing the prevalence of local (oral itch and angioedema) and systemic side effects, including EoE.^28^, ^48^

## 6 CONCLUSION

GI manifestations are a common and significant challenge in OIT for FA. Clinicians must be vigilant in recognizing distinct clinical presentations, ranging from immediate IgE‐mediated reactions to delayed eosinophil‐associated diseases. A shared decision‐making approach, guided by the severity of clinical manifestations and patient priorities, is crucial for managing these conditions. This approach combines dose adjustment with appropriate diagnostic workups, including endoscopy, and targeted pharmacologic therapies. As OIT use becomes more widespread, a deeper understanding of the underlying immunologic mechanisms and the role of factors such as the gut microbiome will be critical for developing personalized protocols that maximize both the safety and efficacy of this transformative therapy.

## AUTHOR CONTRIBUTIONS

Conceptualization: M.V., R.O., M.F. Methodology: M.V., R.O., M.F. Writing – original draft: M.V., M.F. Writing – review and editing: M.V., M.F., M.G. Supervision: L.C., F.C., F.F., S.F.R., M.L., G.L.M., M.M.G., M.G., S.B.

## FUNDING INFORMATION

The authors declare that they did not receive any funding for this study.

## CONFLICT OF INTEREST STATEMENT

M.G. received personal fees from Sanofi and Thermo Fisher Scientific. The authors declare no conflict of interest.

## Data Availability

The data that support the findings of this study are available from the corresponding author upon reasonable request.
